# An Elaborated Signal Model for Simultaneous Range and Vector Velocity Estimation in FMCW Radar

**DOI:** 10.3390/s20205860

**Published:** 2020-10-16

**Authors:** Sergei Ivanov, Vladimir Kuptsov, Vladimir Badenko, Alexander Fedotov

**Affiliations:** Institute of Physics, Nanotechnology and Telecommunications, Peter the Great St.Petersburg Polytechnic University, 29 Polytechnicheskaya str., Saint Petersburg 195251, Russia; vdkuptsov@yandex.ru (V.K.); afedotov@spbstu.ru (A.F.)

**Keywords:** automotive LFMCW radar, radial velocity, lateral velocity, Doppler-frequency estimation, waveform, signal model

## Abstract

A rigorous mathematical description of the signal reflected from a moving object for radar monitoring tasks using linear frequency modulated continuous wave (LFMCW) microwave radars is proposed. The mathematical model is based on the quasi-relativistic vector transformation of coordinates and Lorentz time. The spatio-temporal structure of the echo signal was obtained taking into account the transverse component of the radar target speed, which made it possible to expand the boundaries of the range of measuring the range and speed of vehicles using LFMCW radars. An algorithm for the simultaneous estimation of the range, radial and transverse components of the velocity vector of an object from the observation data of the time series during one frame of the probing signal is proposed. For an automobile 77 GHz microwave LFMCW radar, a computer experiment was carried out to measure the range and velocity vector of a radar target using the developed mathematical model of the echo signal and an algorithm for estimating the motion parameters. The boundaries of the range for measuring the range and speed of the target are determined. The results of the performed computer experiment are in good agreement with the results of theoretical analysis.

## 1. Introduction

In modern automobiles, microwave active locating systems are key elements of the widely adopted high-performance intelligent technologies [1,2]. The use of car microwave radars as part of the Advanced Driver Assistance System (ADAS) ensures the most comfortable and safe movement of the vehicle, and their use as part of the automatic control system provides a completely autonomous (unmanned) mode of vehicle movement.

The ever-increasing requirements for the advanced ADAS systems of the “intelligent” car pose challenges for the solution of which fundamentally different approaches are required. This includes the use of more sophisticated quasi-real-time signal processing algorithms, alternative modulation of sounding radar signals and the development of efficient microwave radar hardware [3,4,5,6]. 

Nowadays, it is generally accepted that for remote radio monitoring of the location and movement of surrounding objects in real time, i.e., simultaneously assessing the speed, range and bearing of targets in automotive applications, it is most effective to use microwave radars with linear frequency modulation (LFMCW) [1,2,3,4,5,6]. In active radar, the target speed is determined by the Doppler frequency shift, and the range is determined by the time delay of the radio signal propagation in the environment [7,8,9]. For automotive LFMCW radars, two main algorithms can be distinguished for simultaneous estimation of target speed and range. The first algorithm is based on a one-dimensional (1D) Fourier transform of the echo signal after it is converted to an intermediate frequency (IF) signal in the quadrature channels of the radar receiver [10,11]. The advantage of this algorithm is the relative simplicity of the technical implementation, the narrow operating frequency band of the radio frequency path and, as a consequence, the low sampling rate of the analog-to-digital converter. A significant disadvantage of the method is the appearance of false radar targets (false alarms) [12]. One of the ways to combat false targets is the use of a segmented structure of a sounding radio signal with variable modulation parameters: chirp duration, frequency deviation and initial generation frequency [13,14,15,16,17]. The creation of a segmented structure of the sounding radio signal can significantly reduce the likelihood of false targets. Phantom targets are eliminated in the process of monitoring the radar situation using tools of intelligent data analysis technologies (Data Mining), such as fuzzy logic, neural networks and Kalman filtering [18,19]. Solving the problem of false radar targets inevitably leads to an increase in the duration of observation and data analysis, which limits the application area of LFMCW radars that use the one-dimensional (1D) Fourier transform algorithm.

The second method of simultaneous estimation of target speed and range is based on the use of two-dimensional (2D) Fourier transform [20,21,22]. It is easy to show that the dynamic range of measured velocities and distances using this method is proportional to the sampling rate *f_s_* of the analog IF signal:

(1) $$f_{s}=8f_{0}\frac{R_{max}}{\delta R}\frac{V_{R\;max}}{c}=4f_{D\;max}\frac{R_{max}}{\delta R},\;\;\;$$

where *R_max_* and *δR*—maximum range and range resolution respectively, *c*—speed of light, *V_R_* *_max_*—maximum radial component of target velocity, *f_D max_*—Doppler frequency shift, that corresponds to maximum velocity *V_R max_*, and *f*_0_—the initial frequency of the radiated chirp radio signal. 

With typical parameter values for vehicle motion control tasks *R_max_*/*δR* = 300, *f*_0_ = 80 GHz and *V_R max_* = 300 km/h, the frequency *f_s_* reaches a value of more than 50 MHz, which presents certain difficulties for the technical implementation of LFMCW radars. To separate the “fast” and “slow” time scales in the received radio signal, which is necessary for an unambiguous estimate of the velocity, the condition for limiting the chirp duration T*_c_* in a sequence of N chirps of one frame of the sounding signal must be met [23]:

(2) $$T_{c}\leq \frac{1}{2f_{D\;max}},$$

(3) N ≥2[log2(VR maxδVR)].

Relation Equation (3) defines the lower bound on the duration of a frame to achieve a given speed resolution *δV_R_*, square brackets mean rounding up to the nearest integer.

In References [23], a model of the sounding signal and methods for estimating the radial velocity are proposed for the case when Equation (2) is not satisfied. The recommended displacement of the initial frequency of neighboring chirps with subsequent analysis of the phase or frequency relationships of two sequences of chirps with different parameters makes it possible to determine the Doppler frequency shift of the target in the Nyquist zones above the first. However, a rigorous mathematical analysis and self-noise accounting of the radar receiving system, carried out in Reference [24], showed that the resulting estimate of the radial velocity can be ineffective and unstable. 

In recent years, leading manufacturers of microwave transceivers for LFMCW radars have been supplying equipment to enable future ADAS (advanced driver-assisted system) safety features, which are highly integrated, high-performance and cost-effective packaged SoC (system on chip) solutions [25]. The sampling rate *f_s_* of analog-to-digital converters of automobile radars reaches 40 MHz and increasing it to 100–150 MHz is not a difficult technical problem. Thus, the functionality of modern microwave transceivers in a wide range of changes in the range and speed of the object is not limited to meeting the requirements of Equations (2) and (3). In this case, an adequate mathematical description of the reflected radio signal, free from the limitations and assumptions adopted in the “classical” mathematical model, plays a key role in the development of algorithms for estimating the range and speed of the target.

As a rule, the signal reflected from the target and arriving at the input of the radar receiving module is written in the form:

(4) u(t,R) = s(t−τ(t)),

where the time delay *τ*(*t*) of propagation of a radio signal in an environment with a speed *c* is determined from the solution of the nonlinear equation [26,27]:

(5) τ(t) = 2R(t−τ(t))c,

whose approximate solution for *c*<<V has the form:

(6) τ(t) = 2∥R+Vt∥c ≈ 2(R+VRt)c  .

Here, *R* is the radius vector that determines the position of the target at the initial time *t* = 0. When deriving Equation (5), the three-dimensional Galilean transformation of space-time for the inertial system is used. There are known special cases [28] of solving active location problems, when the results of calculating the Doppler frequency shift using the Lorentz space-time transformation [29,30] coincide with similar results following from the Galilean transformation. However, in this paper, in the generalized mathematical model of the echo signal, we use the three-dimensional vector form of the equations of the Lorentz transformations, as a more “physical” and adequate mathematical description of the propagation of a space-time sounding radio signal.

The use of linear approximation Equation (6) to calculate the norm of the vector ||***R*** + ***V****t*|| at a high-speed *V_R_* and a small distance *R* to the target leads to a significant error in estimating the values *V_R_* and *R* for any algorithm for processing the input radio signal. The linear approximation Equation (6) does not take into account the transverse (lateral) movement of the target with a speed ***V****_TR_*. At the same time, the ***V****_TR_* velocity component is very important for determining the direction and overall speed of a radar target, and in the approximation Equation (6), can be determined only from the results of sequential processing of several frames [31] of the input radio signal. In References [32,33,34], to determine the transverse velocity component based on the analysis of data obtained over the time interval of one frame (frame) of a radio signal, parallel processing of echo radio signals reflected from several reflex zones of a radio monitoring object is proposed. This method makes rather stringent technical requirements for the characteristics of LFMCW radars, in particular, the angle resolution and the computational performance of the data processor.

In Reference [34], an algorithm for estimating the transverse velocity component based on the results of processing one frame (frame) of a radio signal is considered. However, estimates of the *V_TR_* value were obtained under the assumption of “strictly” lateral movement of the radar target relative to the LFMCW radar, which limits the usage of the proposed algorithm in practical applications.

When solving problems of radar monitoring of vehicles, as a rule, an approximate calculation of the time delay is used *τ*(*t*):

(7) τ2(t)≈τ02=4R2c2,

which is another important factor limiting the permissible range of speed and range measurements. In References [35,36,37,38,39], a refined mathematical model of the reflected radio signal *u*(*t*,*R*) is proposed in which the delay is considered as a function of the slow time scale *τ*(*kT_c_*). In the development of algorithms for estimating the speed and range of a target, in this case, methods of focusing, contrast optimization and discrete polynomial transformation of the signal phase are used [40]. In the mentioned works [35,36,37,38,39], the transverse component of the ***V****_TR_* velocity is not taken into account.

The purpose of this study was to develop a software simulator that implements a signal that is identical to the real echo signal of the LFMCW radar for a single-point reflective radar target. The goal was also to develop an algorithm for estimating the range and velocity vector of a vehicle, including its transverse component, and to conduct a comparative computational experiment.

The structure of the paper is as follows. Section 2 of the paper is devoted to consideration of a generalized mathematical model of the reflected signal from a moving object based on the vector Lorentz transformation of coordinates and time. In Section 3, the spatio-temporal (ST) structure of the LFMCW radar echo signal, taking into account the transverse component of the radar target velocity vector, and considering the mathematical model of the signal at the I-Q output of the receiver’s quadrature channels, is described. In Section 4, the derivation of the relationships for the simultaneous assessment of the distance and the velocity vector of an object, and also analyses of the permissible working range of measurement of motion parameters, are presented. In Section 5, the results of a computational experiment for estimating the parameters *R*, *V_R_* and *V_TR_* are analyzed. Finally, the concluding remarks and future research directions are presented in Section 6.

## 2. Universal Mathematical Model of the Space-Time Echo Signal at an Active Radiolocation of a Moving Target

As mentioned above, the Doppler effect plays a decisive role in assessing the range and speed of targets in automotive LFMCW radars. The mathematical description of the Doppler effect in a wide range of velocities and distances to the target has its own distinctive features that must be taken into account when assessing motion parameters using automotive LFMCW radars. In this section, a mathematical model of the space-time echo-signal with active radar of a moving target, taking into account the Doppler effect and in the absence of restrictions on the magnitude of the speed and range of a single target, is discussed. This makes it possible to use this model to implement a software simulator of the reflected signal when carrying out a computational experiment and testing the performance of algorithms for estimating the parameters of an object’s motion.

It is assumed that the radar antenna is matched in polarization with the radio signal, which is vector electromagnetic radiation. In this case, the polarization effects can be disregarded and the electromagnetic radiation is described by a scalar complex-valued function *u*(*t*,***R***) of two variables—time, *t,* and radius-vector, ***R***, specifying the position of the observation point in space relative to an arbitrary point on the antenna aperture, **Σ**. We consider the case of one-point reflection of electromagnetic radiation, i.e., a radar target as a reflector has a dimension of 0.

**Figure 1:**
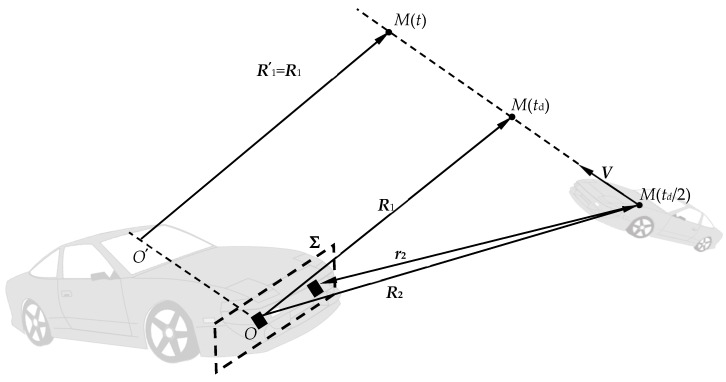
Coordinate system in the task of active location of a moving target.

We will assume that on the time interval *T*, equal to the signal observation time during one frame, the target *M* moves uniformly and rectilinearly at a speed, ***V***, relative to the receiving-transmitting aperture, **Σ**, of the automobile LFMCW radar antenna. Consider two inertial reference systems [41] (Figure 1): the first fixed system (***R***,*t*) is associated with the antenna aperture with the origin of coordinates *O* at the reference fixed point **Σ**, and the second is the movable system (***R***′,*t*′), “tightly” associated with the target *M* (the origin is at point *O*′). For the initial reference time, we will take the moment of the beginning of observation of the emission of the sounding signal. The origin *O*′ of the moving system of coordinates is chosen so that when *t* = 0, it coincided with the beginning of the fixed coordinate system *O*.

Let the probing signal be emitted from the reference point *O* and its form is determined by the complex-valued function *s*_0_(*t*). Then, in the coordinate system associated with the aperture **Σ**, the spatio-temporal structure of the sounding signal at an arbitrary point ***R*** outside the near-field zone of the antenna has the form:

(8) $$u_{0}\left(t,R\right)=\frac{C_{0}U\left(t,R\right)}{∥R∥}{\dot{s}}_{0}\left(t-\frac{∥R∥}{c}\right),$$

where *c*—speed of light (group signal velocity in the medium), *U*(*t*,***R***)—signal amplitude, the value of which is determined by the radiation power, antenna gain and its radiation pattern over the field [42,43], and *C*_0_—constant. In general, *U*(*t,**R***) is a function of time and radius vector ***R***. In modern automotive LFMCW radars, a phased array antenna with a flat aperture and the ability to electronically control the directional pattern is used as an emitting structure [42,43].

The calculation of the echo signal at the antenna aperture includes two stages of the space-time transformation of the sounding radio signal Equation (8).

- With the help of Lorentz transformations [29,30], which put together coordinates and time in inertial systems (***R***,*t*) and (***R***′,*t*′), we pass to a moving frame of reference associated with the target. Determine the field strength *u*_0_(***R***′,*t*′) at time *t*′ at an arbitrary point ***R***′. We take into account that in the frame of reference (***R***′,*t*′), the location of the target *M*, regardless of time, is determined as ***R***′ = ***R***_1_, where ***R***_1_ is the position of the target in the system (***R***,*t*) at the time *t_d_* = 2||***R***_2_||/*c*—the time of arrival of the reflected signal at point *O* (Figure 1). Field-induced current at a target location in a moving coordinate system where the target is stationary:

$$i_{t}\left(t^{\prime }\right)=C_{1}u_{0}\left(R^{\prime },t^{\prime }\right)\exp\left[j\phi \left(t^{\prime }\right)\right],$$

where *C*_1_—constant, and *ϕ*(*t*)—phase shift between field and current. This current generates a secondary radiation field determined by the formula:

(9) $$\dot{u}\left(t^{\prime },R^{\prime }\right)=\frac{C_{2}\left(t^{\prime },R^{\prime }\right)}{∥R^{\prime }-R_{1}∥}i_{t}\left(t^{\prime }-\frac{∥R^{\prime }-R_{1}∥}{c}\right),$$

where *C*_2_(*t***, *R***′)—in the general case, the function of time and radius vector ***R***′ and the form of which is determined by the scattering indicatrix of the radar target [44,45].

**Figure 2:**
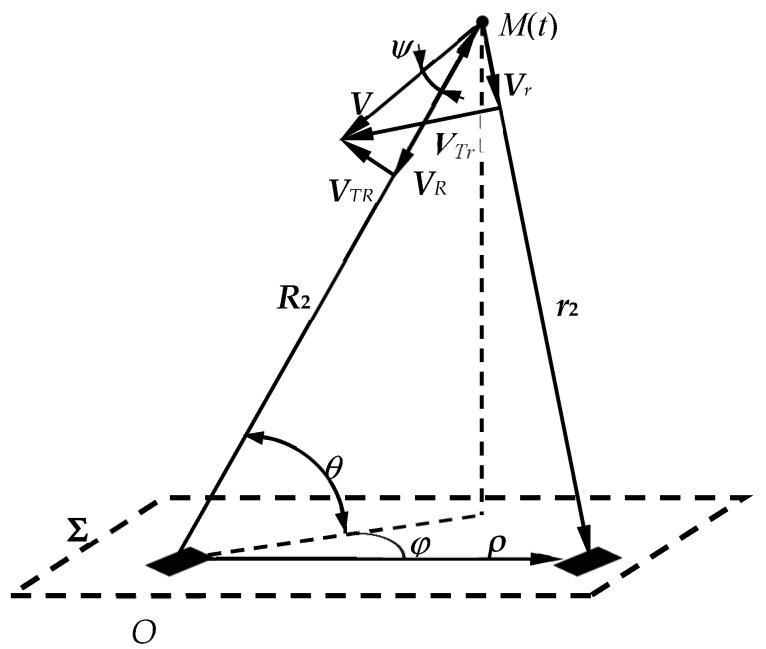
Radial and transverse components of the target velocity vector.

- To determine the space-time structure of the field at the aperture **Σ** of the antenna, the reverse transition to the stationary frame of reference (***R***,*t*) is carried out using the Lorentz transformation. It is convenient to operate with the vector ***R***_2_, which determines the position of the target at the moment of the onset of re-emission, i.e., at time *t* = *t*_d_/2. From Figure 1, it follows that ***R***_1_ = ***R***_2_ + ***V****t*_d_ = ***R***_2_ + ***V****R*_2_/2, and on the antenna aperture, the coordinates of an arbitrary point ***R*** are characterized by the radius vector ***ρ*** and, therefore, ***R*** = ***ρ*** for the region **Σ** (Figure 2). It is advisable to set the vector ***r***_2_ = ***ρ*** − ***R***_2_ as a vector connecting the target with an arbitrary point ***ρ*** of the antenna aperture at time *t* = *t*_d_/2 (see ). It is efficient to decompose the target velocity vector ***V*** into two orthogonal components ***V****_R_* and ***V****_TR_*, one of which, ***V****_R_*, is collinear to the radius vector ***R***_2_, the other, *V_TR_*, is the transverse velocity component (see Figure 2). Also, the decomposition of the velocity vector ***V*** into two orthogonal components is possible with respect to the base radius vector ***r***_2_. In this case, we obtain the components ***V****_r_* and ***V****_Tr_*, respectively (see Figure 2). In Figure 2, *ψ* is the angle between the orthogonal components of the velocity ***V****_R_* and ***V****_TR_*, and the angles *θ* and *φ* characterize the angular position of the target *M* in a spherical coordinate system centered at point *O* and the reference plane **Σ**.

At a speed of the observed objects up to 350 km/h (upper segment of sports cars), the maximum value of the ***V****_max_* speed module is |***V****_max_*| < 2⋅350 = 700 km/h. The maximum duration of one frame of the sounding radio signal usually does not exceed 40 ms. In this case, for 77 GHz Automotive Radar Applications, as the calculations show, the relativistic factor in the Lorentz transformations can be ignored and “truncated” equations can be used:

(10) $$R^{\prime }=r+V\left[\left(\beta -1\right)R\cdot r/V^{2}-\beta t\right]\approx R-Vt;\;\;\;\;\;\;\;R\approx R^{\prime }+Vt^{\prime };\;\;$$

(11) t′≈t−V·Rc2;       t≈t′+V·R′c2,

*V* = ||***V***||; *β* = (1−*V*^2^/*c*^2^)^−1/2^. The dot between the vectors hereinafter means the scalar product of the vectors. 

Calculation of the space-time structure of the echo radio signal *u*(*t*,*r*_2_) at the antenna aperture **Σ** in accordance with the above technique leads to the following expression:

(12) $$u\left(t,r_{2},R_{2},V\right)\;=\;{\dot{U}}_{Rv}s_{0}\left(t-\tau \left(t,r_{2},R_{2},V\right)\right),$$

where the time delay τ(*t*,***r***_2_,***R***_2_,***V***) is equal to:

(13) $$\tau (t,r_{2},R_{2},V)=\frac{V\cdot r_{2}}{c^{2}}-\frac{1}{c}∥r_{2}-V\left(t-\frac{R_{2}}{c}\right)∥-\frac{1}{c}∥\{R_{2}+V\left[t-\frac{R_{2}}{c}-\frac{1}{c}∥r_{2}-V\left(t-\frac{R_{2}}{c}\right)∥\right]\}∥,$$

where *R*_2_ and *r*_2_ are modules (norms) of the corresponding radius vectors. In Equation (13) for *τ*(*t*,***r***_2_,***R***_2_,***V***), the time is counted from the moment the sounding signal begins to be emitted.

Relations Equations (12) and (13) describe a universal mathematical model of the space-time structure of the signal with active location of moving targets using microwave radars. The versatility of the model implies an arbitrary functional time dependence of the sounding signal *s*_0_(*t*), the range of speeds 0 < *V* < 4 km/s and distances *R*, covering the limiting parameters of the movement of existing and future land vehicles. The complex amplitude *U_Rv_* is proportional to the product *U*(*t*,***R***)*C*_2_(***R***′)exp[*jϕ*(*t*)], i.e., is a complex-valued function of the variables *t*, ***R*** and ***R***′. Typically, the form of this function is determined experimentally or by semi-empirical methods. However, for automotive LFMCW radars, further analysis of the mathematical model of the signal reflected from the target space-time structure of the signal is carried out in the approximation *U_Rv_* = const(*t*,***R***,***R***′). The validity of such an approximation follows from the short duration of an individual chirp of the *T*_c_ signal. 

The results obtained refine the results of Reference [41]. The obtained relations Equations (12) and (13) make it possible to implement a software simulator of the reflected signal during a computational experiment. On the basis of the considered universal mathematical model, we will develop a more accurate description of the spatio-temporal structure of the LFMCW radar echo signal, taking into account the transverse component of the radar target velocity vector, and which can be effectively used to implement signal processing algorithms.

## 3. Mathematical Model of Echo Radio-Signal Conversion in the LFMCW Radar Receiver

In this section, the developed general universal mathematical model of the spatio-temporal echo of a signal with active radar in the quasi-relativistic approximation for an arbitrary waveform is used to construct a mathematical model of the reflected radio signal for the case of LFMCW automotive radars. Consideration is carried out in the approximation of a distant location, when the value of the parameter equal to the ratio of the distance the target moves during processing within one frame to the distance to the target is less than one.

**Figure 3:**
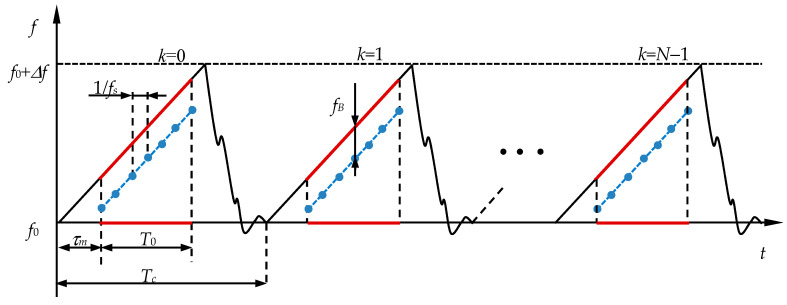
Radio signal of the transceiver. Blue—receiver radio signal, red—the working area of the sounding radio signal.

As noted above, modern automotive radar hardware makes it possible to generate and receive a continuous radio signal *s*_0_(*t*) in the form of a periodic sequence of segments with linear frequency modulation of the signal in Figure 3, with given operating parameters *N*, *f_s_* and *T_c_*, the values of which satisfy constraints Equations (1)–(3). The requirement for the radar range resolution *δR* imposes additional conditions on the frequency deviation Δ*f* and the rate of change of the chirp frequency *α* = Δ*f*/*T_c_*:

(14) Δf≥c2δR;   and αTc≥c2δR.

A mathematical description in the time domain of the sounding radio signal *S_Tr_*(*t*), shown in Figure 3, has the following form:

(15) $$S_{Tr}\left(t\right)=U_{Tr}\cos\left\{2\pi f_{0}\left(t-\left[\frac{t}{T_{c}}\right]T_{c}\right)+\pi \alpha {\left(t-\left[\frac{t}{T_{c}}\right]T_{c}\right)}^{2}{+}_{\phi_{Tr}}\right\}\mathrm{rect}\left(\frac{t-\left[\frac{t}{T_{c}}\right]T_{c}-\tau_{m}}{T_{0}}-\frac{1}{2}\right),$$

where the square brackets operator [∗] means rounding a number down to the nearest integer, rect(*x*)—rectangular function [46], *ϕ_Tr_*—the initial phase of the chirp, *τ_m_*—start of work of the ADC of the receiver within one chirp, and *T*_0_—the working interval of data collection, during which the analog IF signal is converted into a digital code in the radar receiver. In Figure 3, the radio signal of the receiver is highlighted in blue, and the working area of the sounding radio signal is in red. For the received radio signal *S_Rv_*(*t*) in the time domain, we can write:

(16) $$S_{Rv}\left(t\right)=S_{Tr}\left[t-\tau \left(t,r_{2},R_{2},V\right)\right],$$

where the time delay *τ*(*t*,***r***_2_,***R***_2_,***V***) in the general case is determined by the relation Equation (13). In Equation (16) and below, for brevity, the dependence of the signal is on variables ***r***_2_, ***R***_2_, ***V***.

Further conversion of the frequency-modulated radio signal in the receiving path of the radar occurs in accordance with the coherent quadrature demodulation algorithm, in which the microwave signal is converted to the zero-frequency region using mixers in the I and Q quadrature channels. In this case, the signal at the output of the *S_MIX_*(*t*) mixers in the general case can be represented by a complex-valued function:

(17) $${\dot{S}}_{MIX}\left(t\right)=U_{M}\exp\;\left\{\arg\;\left[{\dot{S}}_{Tr}\left(t\right)\right]-\arg\;\left[{\dot{S}}_{Rv}\left(t\right)\right]\right\}={\dot{U}}_{M}\exp\;\left\{\arg\;\left[{\dot{S}}_{Tr}\left(t\right)\right]-\arg\;\left[{\dot{S}}_{Tr}\left(t-\tau \left(t,r_{2},R_{2},V\right)\right)\right]\right\}.$$

For further analysis, it is convenient to represent the current time *t* as the sum of the local time *t_L_* (fast scale) and discrete time *kT_c_* (slow scale):

t=t(tL,k)=tL+τm+[tTc]Tc=tL+τm+kTc,

(18) $$\forall \;\;\left\{t_{L}\in \mathbb{R}:0\leq t_{L}\leq T_{c}\right\}\times \left\{k\in \mathbb{Z}:0\leq k\leq N-1\right\}.$$

In the particular case of a sounding radio signal with linear frequency modulation Equation (15), the signal from the output of the mixer Equation (17), taking into account Equation (18), will have the following form:

(19) $${\dot{S}}_{MIX}\left(t_{L},k\right)={\dot{U}}_{M}\exp\left(j\left\{2\pi f_{0}\left(t_{L},k\right)+2\pi \alpha \tau \left(t_{L},k\right)\left[t_{L}+\tau_{m}-\frac{\tau (t_{L},k)}{2}\right]+\phi_{Tr}-\phi_{Rv}\right\}\right)\mathrm{rect}\left(\frac{t_{L}}{T_{0}}\right).$$

Relations Equations (13) and (15)–(19) describe a universal mathematical model for transforming the spatio-temporal structure of the signal during active location of moving targets in microwave radars using a continuous sounding radio signal Equation (15) with linear frequency modulation. The range of permissible target speeds and ranges for this echo radio-signal model makes it applicable for analyzing the operation and prototyping of LFMCW radars that locate existing and future ground vehicles. However, the complex, nonlinear nature of the universal mathematical model of the echo radio signal makes it ineffective in the development of promising quasi-real-time systems for estimating the parameters of the movement of vehicles in the current state of development of software and hardware. Let us consider an approximate mathematical model of an echo radio signal, which makes it possible to significantly simplify the algorithms for processing and analyzing the input data of the radar.

Modes of operation of automotive LFMCW radars are subdivided into near and far location of the workspace. For near location (e.g., when the vehicle is parking), a short range and target speed are typical. For far radiolocation, the distance to the target ranges from several meters to several hundred meters, and the target speed ranges from fractions of a meter per second to 200 m/s. The further developed mathematical model of the spatio-temporal structure of the signal can be effectively used for the mode of long-range location of the working space with an automobile LFMCW radar. We take into account that the results of radar imaging should be promptly visualized on a user monitor, and the signal processing time should not exceed 20–40 m/s. In the FMCW long-range radar mode, the distance the target moves during processing usually does not exceed the distance to the target, and the Maclaurin power series expansion can be used for the vector norm ||***R*** − ***V****t*|| [46]:

(20) $$∥R-Vt∥=\sqrt{R^{2}-2V\cdot Rt+{\left(Vt\right)}^{2}}=R\sqrt{1-\frac{2V\cdot Rt}{R^{2}}+\frac{{\left(Vt\right)}^{2}}{R^{2}}}\;=R\overset{\infty }{\underset{n=0}{\sum }}\frac{{\left(-1\right)}^{n}\left(2n\right)!}{\left(1-2n\right){\left(n!\right)}^{2}\left(4^{n}\right)}{\left(\frac{{\left(Vt\right)}^{2}}{R^{2}}-\frac{2V\cdot Rt}{R^{2}}\right)}^{n}\;\approx R-V_{R}t+\frac{V_{TR}^{2}t^{2}}{2R}\left(1+\frac{V_{R}t}{R}+\frac{V_{R}^{2}t^{2}}{R^{2}}+O\left(\frac{V^{3}t^{3}}{R^{3}}\right)\right),$$

where *V_R_*—norm of velocity vector projection onto radius vector ***R,*** (*V_R_* = ***V***⋅***R***). Similarly, we can use ***V****_r_*—the projection of the velocity on the radius vector ***r***, as well as the corresponding orthogonal components ***V****_TR_* and ***V****_Tr_* (see Figure 2). 

The domain of convergence of a power series Equation (20):

(21) $$\left|\frac{{\left(Vt\right)}^{2}}{R^{2}}-\frac{2V\cdot Rt}{R^{2}}\right|=\left|\frac{V^{2}t^{2}}{R^{2}}-\frac{2V_{R}t}{R}\right|\leq 1,$$

which must be taken into account when assessing the range and speed measurement limits of a radar target in this approximation. In microwave LFMCW radars of the millimeter wavelength range, the dimensions of the antenna aperture are significantly less than the minimum specified distance to the target. In this case, for the vector velocity, ***V,*** projections norms with high accuracy *V_TR_* = *V_Tr_* and *V_R_* = *V_r_*.

Power series expansion Equation (20) of the norm of the vector ||***R*** − ***V****t*|| accurate to the third order of smallness relative to *Vt*/*R* allows to find an approximate expression for calculating the time delay *τ*(*t*,***r***_2_,***R***_2_,***V***). From the general relation Equation (13), we obtain:

(22) $$\tau (t,r_{2},R_{2},V)\approx \tau_{s}\left(t_{L},k\right)\;\;\;\;\;\;=\tau_{R}-\frac{1}{c}2V_{R}(t_{L}+\tau_{m}+kT_{c})-\frac{\rho }{c}cos\theta \;\;\;\;\;\;+\frac{V_{TR}^{2}}{cR_{2}}{\left[t_{L}+\tau_{m}+kT_{c}\right]}^{2}\left[1-\frac{V_{R}(t_{L}+\tau_{m}+kT_{c})}{R_{2}}+\frac{V_{R}^{2}{\left(t_{L}+\tau_{m}+kT_{c}\right)}^{2}}{R_{2}^{2}}\right]\;\;\;\;\;\;+\frac{2V_{R}}{c}\tau_{R}=\tau_{R}-\frac{1}{c}2V_{R}(t_{L}+\tau_{m}+kT_{c})-\frac{\rho }{c}cos\theta +\Delta \tau_{s}\left(t_{L},k\right)$$

where *θ*—the angle of inclination of the radio wave front to the antenna aperture (see Figure 2), and *τ_R_* = 2*R*_2_/*c*—propagation delay of the sounding radio signal, determined by the doubled distance to the target at the time of its re-emission. 

The first three terms in Equation (22) are the propagation delay of the sounding radio signal, considered in the traditional approach to the theoretical analysis of the characteristics of the automotive LFMCW radar. The dependence of the third term in Equation (22) on the **ρ** coordinate of the receiving element at the antenna aperture makes it possible to determine the direction to the radiation source—the target bearing when using a phased antenna array in the radar. The additional term Δ*τ_s_*(*t_L_*,*k*) makes a significant contribution to the propagation delay of the sounding radio signal as the transverse velocity component increases and the distance to the target decreases.

A mathematical model of the complex-valued signal at the I-Q output of the quadrature channels of the LFMCW radar receiver, which takes into account the transverse component of the target velocity and, accordingly, retains its rigor in a wide range of changes in the speed and range to the observed target, is obtained by substituting the found value of the delay *τ_s_*(*t_L_*,*k*) Equation (22) into Equation (19), which determines the mixer signal in the general case:

(23) $${\dot{S}}_{MIX}\left(t_{L},k\right)=U_{M}\exp\left\{2\pi j\left[\alpha \tau_{R}+\frac{2V_{R}}{c}f_{0}+\frac{2V_{R}}{c}\alpha kT_{c}\;+\frac{V_{TR}^{2}}{cR_{2}}kT_{c}\left(1+\frac{V_{R}kT_{c}}{R_{2}}\right)\left(2f_{0}+\alpha kT_{c}\right)\right]t_{L}\;+2\pi jkT_{c}\left[\frac{2V_{R}}{c}f_{0}-\tau_{R}\frac{2V_{R}}{c}\alpha +\tau_{m}\frac{2V_{R}}{c}\alpha +\frac{V_{TR}^{2}}{cR_{2}}kT_{c}\left(1+\frac{V_{R}kT_{c}}{R_{2}}\right)f_{0}\right]+\Delta \phi \right\}\mathrm{rect}\left(\frac{t_{L}}{T_{0}}\right),$$

here, Δ*ϕ*—component of the phase of the mixer signal, in the first approximation independent of the time *t* in the observation interval *T*:

$$\Delta \phi =\phi_{Tr}-\phi_{Rv}+2\pi \left[f_{0}\tau_{R}+\alpha \left(\tau_{R}-2\tau_{m}\right)\tau_{m}\right].$$

For the physical interpretation of the results obtained, it is advisable to introduce the phase *Φ*(*t_L_*,*kT*_c_) of the complex-valued signal of the *S_MIX_*(*t_L_*,*k*) and rewrite Equation (23) in the following form:

(24) $${\dot{S}}_{MIX}\left(t_{L},k\right)=U_{M}\exp\left[j\Phi \left(t_{L},kT_{c}\right)\right]\;\mathrm{rect}\left(\frac{t_{L}}{T_{0}}\right)\;\;\;\;\;\;\;\;\;\;\;\;\;\;\;=U_{M}\exp\left\{j\left[\Phi_{B}\left(t_{L},kT_{c}\right)+\Phi_{V}\left(kT_{c}\right)\right]+\Delta \phi \right\}\mathrm{rect}\left(\frac{t_{L}}{T_{0}}\right)\;\;\;\;\;\;\;\;\;\;\;\;\;\;\;\;\;\;\;\;\;=U_{M}\exp\left\{2\pi j\left[f_{B}\left(kT_{c}\right)t_{L}+\int_{0}^{kT_{c}}f_{V}\left(kT_{c}\right)d\left(kT_{c}\right)\right]+\Delta \phi \right\}\mathrm{rect}\left(\frac{t_{L}}{T_{0}}\right),$$

where *f_B_*(*kT_c_*)—instantaneous frequency of the complex-valued signal *S_MIX_*(*t_L_*,*k*), in the technical literature called the beat frequency:

(25) $$f_{B}\left(kT_{c}\right)=\frac{\partial \left[\Phi_{B}\left(t_{L},kT_{c}\right)/2\pi \right]}{\partial t_{L}}=\alpha \tau_{R}+\frac{2V_{R}}{c}f_{0}+\frac{2V_{R}}{c}\alpha kT_{c}+\frac{V_{TR}^{2}}{cR_{2}}kT_{c}\left(1+\frac{V_{R}kT_{c}}{R_{2}}\right)\left(2f_{0}+\alpha kT_{c}\right)\;=\;\alpha \tau_{R}+\frac{2V_{R}}{c}f_{0}+\Delta f_{B}\left(kT_{c}\right).$$

In Figure 3, *f_B_* value is shown as the current difference between the frequencies of the sounding and echo radio signals within the duration of each *k* chirp of the radio signal. The one-dimensional Fourier transform makes it easy to calculate the beat frequency *f_B_*(*kT_c_*) as a function of *k*. The term Δ*f_B_*(*kT_c_*) in Equation (23), which is additional in comparison with the classical method of analysis, is the result of using a more rigorous mathematical model of radio signal transformation in the radar receiver. A non-trivial conclusion is the nonlinear dependence of the beat frequency on the chirp number *k*, which must be taken into account when evaluating the parameters *R* and *V* at a large value of the transverse velocity and short distances to the target.

The Doppler frequency shift *f_V_*(*kT_c_*) in Equation (23) is described by the formula:

(26) $$f_{V}\left(kT_{c}\right)=\frac{\partial \left[\Phi_{V}\left(kT_{c}\right)/2\pi \right]}{\partial \left(kT_{c}\right)}=\frac{2V_{R}}{c}f_{0}-\tau_{R}\frac{2V_{R}}{c}\alpha +\tau_{m}\frac{2V_{R}}{c}\alpha +\frac{2V_{TR}^{2}}{cR_{2}}kT_{c}\left(1+\frac{3V_{R}kT_{c}}{2R_{2}}\right)f_{0}=-\frac{2V_{R}}{c}f_{0}+\Delta f_{V}\left(kT_{c}\right),$$

where the term Δ*f_V_*(*kT_c_*) refines the proposed mathematical model of radio signal conversion. The dependence of the Doppler frequency shift *f_V_*(*kT_c_*) on the slow time scale *kT_c_* makes the traditional method of estimating the target velocity based on the 2D Fourier transform ineffective. The signal of the mixer *S_MIX_*(*t_L_*,*k*) as a function of time *kT*_c_ with a strict mathematical description belongs to the class of frequency-modulated signals and, therefore, is not narrowband and the definition of the parameter *V* is not unambiguous. The spectrum width increases at a high transverse speed and small target ranges, which leads to an error in determining the parameters *R* and *V*.

The obtained relations Equations (23)–(25), which determine the temporal structure of the received signal by the LFMCW radar, make it possible to develop an algorithm for estimating the motion parameters *V_R_*, *V_TR_* and *R*_2_.

## 4. Estimation of the Range and Velocity Vector of a Radar Target: Working Range of Parameters’ Measurement

In this section, based on the analysis of the mathematical model of the complex-valued signal at the output of the I-Q quadrature channels of the LFMCW radar receiver, an algorithm is proposed for estimating such motion parameters as the radial and transverse components of the velocity, as well as the distance to the radar target. In addition, the boundaries of the range of variation of the variables *V_R_*, *V_TR_* and *R*_2_ are calculated, where the proposed algorithm remains operational.

Let us write the phase *Φ*(*t_L_*,*kT*_c_) of the complex-valued signal of the mixer *S_MIX_*(*t_L_*,*k*) Equation (23) as a third-degree polynomial in the variable *kT*_c_:

(27) $$\Phi \left(t_{L},kT_{c}\right)=2\pi \left[f_{B}\left(kT_{c}\right)t_{L}+a_{0}+a_{1}kT_{c}+a_{2}{\left(kT_{c}\right)}^{2}+a_{3}{\left(kT_{c}\right)}^{3}\right],$$

the coefficients of the polynomial (*a*_0_, *a*_1_, *a*_2_, *a*_3_) are determined from the relation (23):

(28) $$a_{0}\equiv \Delta \phi ;\;\;\;\;\;\;a_{1}=\frac{2V_{R}}{c}f_{0}\left(1-\frac{\alpha \tau_{R}}{f_{0}}+\frac{\alpha \tau_{m}}{f_{0}}\right);\;\;\;\;\;\;a_{2}=\frac{V_{TR}^{2}}{cR_{2}}f_{0};\;\;\;\;\;\;a_{3}=\frac{V_{TR}^{2}}{cR_{2}}\frac{V_{R}}{R_{2}}f_{0}.$$

In accordance with the developed mathematical model for converting the input signal in the radar receiver, the algorithm for estimating the range and speed of a radar target includes the following stages:

(1) Using the Fourier transform, the beat frequency *f_B_*(*k*) and the phase *Φ*(*f_B_*,*k*) of the complex-valued spectral components of the signal of the receiver quadrature channels are calculated at each *k*-th local time interval ∀ {*t_L_* ∈ℝ: 0 ≤ *t_L_* ≤ *T*_c_} in a sequence of *N* chirps of the radio signal. The spectral component corresponds to the target in the radar monitoring area.
(2) The obtained time series of phase samples *Φ*(*f_B_*,*k*) of the selected spectral component (target) is approximated by a polynomial of the third degree in the variable *kT*_c_. As a result of the approximation procedure, we obtain estimates for the coefficients (*a*_0_, *a*_1_, *a*_2_, *a*_3_) of the polynomial.
(3) On the basis of the obtained estimates of the coefficients (*a*_0_, *a*_1_, *a*_2_, *a*_3_) together with the estimate of the beat frequency *f_B_*(*k*), the estimates of the motion parameters—the velocity vector and the distance to the target—are calculated.

The solution of the system of joint Equations (23), (25) and (27) leads to the following formulas for estimating the parameters of the movement of a radar target *R* and *V*:

Target distance estimation:

(29) $${\hat{R}}_{2}\left(k\right)=\frac{c}{2\alpha }\cdot \frac{{\hat{f}}_{B}\left(k\right)-\frac{{\hat{a}}_{1}}{2\pi }\left(1+\frac{\alpha kT_{c}}{f_{0}}\right)\left(1-\frac{\alpha \tau_{m}}{f_{0}}\right)-\frac{{\hat{a}}_{2}}{2\pi }\left(2+\frac{\alpha kT_{c}}{f_{0}}\right)kT_{c}}{1+\frac{{\hat{a}}_{1}}{2\pi }\left(1+\frac{\alpha kT_{c}}{f_{0}}\right)\frac{1}{f_{0}}},$$

hereinafter, the diacritical mark of the cap (circumflex) above the corresponding parameter means the estimate of this parameter obtained as a result of data processing according to the above algorithm.

Estimation of the radial component of the target’s velocity:

(30) $${\hat{V}}_{R}\left(k\right)=\frac{c}{2f_{0}}\cdot \frac{{\hat{a}}_{1}}{2\pi \left(1-\frac{2\alpha {\hat{R}}_{2}\left(k\right)}{cf_{0}}+\frac{\alpha \tau_{m}}{f_{0}}\right)};$$

Estimation of the transverse component of the target speed:

(31) $${\hat{V}}_{TR}\left(k\right)=\sqrt{\frac{{\hat{a}}_{2}}{2\pi }\cdot \frac{c{\hat{R}}_{2}\left(k\right)}{f_{0}}}.$$

If the estimates of the coefficients {*a*_0_, *a*_1_, *a*_2_, *a*_3_} of the polynomial and the beat frequency *f_B_*(*k*) coincide with the true values of the parameters, the estimates of the target range and velocity components will also be equal to their true values. In this case, the estimates do not depend on the chirp number *k*. If the error of estimates {*a*_0_, *a*_1_, *a*_2_, *a*_3_} and *f_B_*(*k*) is not zero, then the algorithm for estimating the range and speed of the radar target should include the stage of calculating the sample mean of the corresponding estimates of the motion parameters.

Expansion of the range of measured motion parameters through the use of the developed algorithm for estimating the range and speed, in comparison with estimates based on traditional algorithms, determines the practical significance of the results obtained. Let us analyze the working range of motion parameters’ measurement. The mathematical model of radio signal transformation, which takes into account, among other things, the transverse component of the velocity, is based on the necessary condition for the vector of measured parameters **θ** = {*V_R_*, *V_TR_*, *R*_2_} belonging to the three-dimensional space **Θ^(3^^)^**:

(32) $$\Theta^{\left(3\right)}\;=\Theta_{1}^{\left(3\right)}\cap^{\;}\Theta_{2}^{\left(3\right)};$$

(33) $$\Theta_{1}^{\left(3\right)}=\Theta_{11}^{\left(3\right)}\cup^{\;}\Theta_{12}^{\left(3\right)}\;=\left\{\theta :\Delta f_{B}\left(NT_{c}\right)T_{c}\geq 1,\theta \;\in \;{\mathbb{R}}^{3}\right\}\cup^{\;}\left\{\theta :\int_{0}^{NT_{c}}\Delta f_{V}\left(NT_{c}\right)d\left(NT_{c}\right)\geq 1,\theta \;\in {\mathbb{R}}^{3}\right\};$$

(34) $$\Theta_{2}^{\left(3\right)}=\left\{\theta :\Phi_{V}\left[\left(k+1\right)T_{c}\right]-\Phi_{V}\left(kT_{c}\right)\leq \pi ,\;\forall \;k\in \left[0,\;N\right],\theta \;\in \;{\mathbb{R}}^{3}\right\},$$

here, the signs ∪ and ∩ denote the union and intersection of two sets, respectively. 

In relation Equation (33), the belonging of the point **θ** to the region **Θ^(^**^3)^_11_ is determined by the noticeable influence of the correction Δ*f_B_*(*kT_c_*) on the beat frequency of the radio signal *f_B_*(*kT_c_*), calculated on the local time interval [0 ≤ *t_L_* ≤ *T*_c_]. The belonging of point **θ** to the second region **Θ^(^**^3)^_12_ is determined by the noticeable influence of the correction Δ*f_V_*(*kT*_c_) on the interval of the slow time scale [0 ≤ *kT*_c_ ≤ *NT*_c_].

For correct use of the method of two-dimensional Fourier transform [19,20,21] and an unambiguous estimate of the velocity vector of a radar target, it is necessary that the phase change *Φ_V_*(*kT*_c_), calculated on the local time interval [0 ≤ *t_L_* ≤ *T*_c_], does not exceed the value π. This requirement determines that the point **θ** belongs to the three-dimensional subspace **Θ^(^**^3)^_2_ of the measured parameters {*V_R_*, *V_TR_*, *R*_2_} in the relation Equation (34).

When constructing a mathematical model of radio signal transformation Equations (23)−(26), it is necessary to fulfill a number of conditions, such as the convergence of the Maclaurin power series expansion Equation (20) and the unambiguity of the parameter estimates Equations (1)−(3). The requirements for LFMCW radar design to the radar resolution in range *δR* and speed *δV*, maximum and minimum measured range *R_max_*, *R_min_* and speed *V_max_*, *V_min_* make the task of determining the boundaries of the region **Θ^(^**^3)^ a multiparameter problem. 

Relations Equations (20) and (32)−(34) lead to the following system of inequalities as necessary conditions determining the boundaries of the domain **Θ^(^**^3)^:

(35) $$\{\begin{array}{c} \frac{2V_{R}NT_{c}}{R_{2}}+\frac{V_{TR}^{2}N^{2}T_{2}^{2}}{R_{2}^{2}}\leq 1;\\ \frac{V_{TR}^{2}N^{2}T_{2}^{2}}{cR_{2}}\cdot \frac{V_{R}NT_{c}}{R_{2}}f_{0}<1\;\;\left(more\;strictly\;\;0.3\right);\\ \frac{V_{TR}^{2}N^{2}T_{2}^{2}}{cR_{2}}f_{0}\geq 1;\\ V_{R}+\frac{V_{TR}^{2}}{R_{2}}NT_{c}<V_{max}. \end{array}$$

The first two inequalities in Equation (35) follow from the conditions for the convergence of the Maclaurin power series Equation (20) and the truncation of terms higher than the second order of smallness in the expansion of the norm of the vector ||***R*** − ***V****t*||. The third Equation (35) defines the lower bound for *V_TR_min_*:

(36) $$V_{TR\_min}=\frac{1}{NT_{c}}\sqrt{\frac{cR_{2}}{f_{0}}},$$

where the contribution of the transverse velocity component to the estimation of the parameters of the movement of the radar target must be taken into account in order to achieve the specified parameters of the resolution *δVδR* in range and speed. 

**Figure 4:**
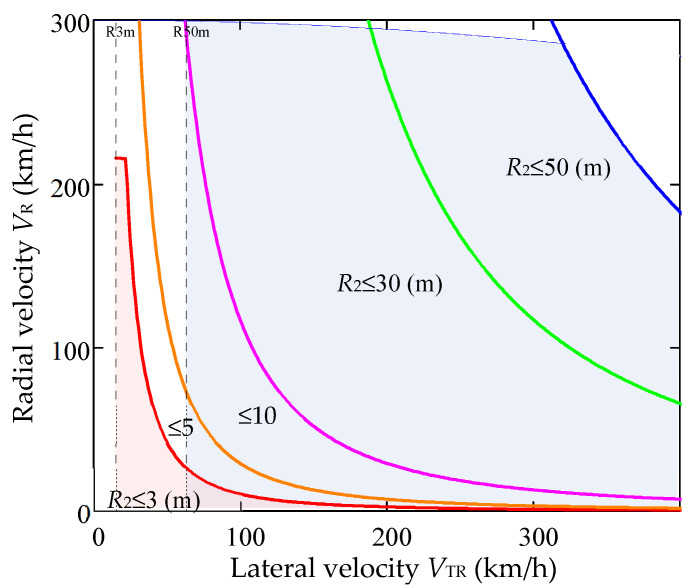
The limits of efficiency of the algorithm for estimating the parameters of the target movement.

In Figure 4 in the *V_TR_*-*V_R_* plane, the curves of the contour of the section by the plane of constant fixed range *R* of the three-dimensional space **Θ^(^**^3)^ are presented, where the efficiency of the developed algorithm for estimating the parameters of the target movement is preserved. Points belonging to **Θ^(^**^3)^ lie between the boundary *V_TR_min_* and the curve of the section contour, with a given value of the range *R*, which is determined by Equation (36). 

For example, in Figure 4, 2D-sections of the region **Θ^(^**^3)^ are selected for the parameter values *R* = 3 m, *V*1*_TR___min_* = 15.6 km/h и *R* = 50 m, *V*2*_TR_min_* = 63.8 km/h. We must note that points in space **Θ^(^**^3)^ are the set of values of the measured parameters {*V_R_*, *V_TR_*, *R*_2_}, the variance and bias of the estimate of which, based on the traditional 2D-FFT (Fast Fourier Transform) algorithm, can have a significant value. 

The analysis of the graphs shown in Figure 4 shows that the operability area of the algorithm for estimating the motion parameters depends not only on the norm of the general velocity vector ||***V***||, but also on the ratio of the components of the velocity vector, that is, on the angle *ψ* of the angle between the velocity vector ***V*** and the radius-vector ***R***_2_ (see Figure 2). For example, with *ψ* = 45° and *V_TR_* = *V_R_* = 200 km/h (total speed *V* = 283 km/h), the lower limit of the range when estimating *V_TR_* with a given speed resolution *δV* = 1 km/h is 45 m. In this case, all three conditions Equation (35) are satisfied (the second with a more stringent requirement, 0.3).

The estimates of the range and velocity vector of a radar target proposed in this section, as well as the calculation of the operating range for measuring the parameters *V_R_*, *V_TR_* and *R*_2_, require experimental verification of the results obtained. The next section discusses the results of a computational experiment using the software simulator developed in the first section of the paper.

## 5. Computational Experiment Results and Discussion

To check the obtained results of theoretical analysis, we carried out a computational experiment simulating the operation of the LFMCW radar in the mode of estimating the movement parameters *V_R_*, *V_TR_* и *R*_2_ of a vehicle. Provided that the intrinsic noise and instability of the parameters of the radar receiving system, as well as the multiple reflections of the probing signal and in the approximation of a single-point reflective target, are neglected, the software simulator implements a signal that is identical to the real echo signal at the output of the quadrature channels of the LFMCW radar receiver. It can be used to test the effectiveness of the developed algorithm for estimating the range and velocity vector of the vehicle. In this sense, carrying out computer modeling using this signal simulator can be called a computational experiment. The parameters *V_R_*, *V_TR_* and *R*_2_ were estimated in accordance with the algorithm developed above Equations (29)–(31). 

The tasks of the computational experiment were as follows:

- For the developed algorithm, in the operating range of variation of *V_R_*, *V_TR_* and *R*_2_, the determination of the parameter estimation error at different values of the angle *ψ* between the radial *V_R_* and transverse *V_TR_* components of the velocity.
- Comparison of the error in estimating the range and speed obtained using the developed algorithm and the traditional method based on two-dimensional FFT.
- Determination of the limits of the working range for measuring the parameters *V_R_*, *V_TR_* and *R*_2_ as a result of a computational experiment and comparison of the obtained data with theoretical calculations of the fourth section.
- Carrying out a computational experiment for the physical interpretation of the results.

During the computational experiment, the following initial values of the parameters were used:

(1) The maximum sampling rate of an analog signal is about 55 MHz,
(2) Upper limit of range measurement—*R_max_* = 400 m,
(3) Range resolution—*δR* = 1.6 m,
(4) Upper limit of speed measurement—*V_max_* = ±300 km/h,
(5) Speed resolution—*δV* = 1 km/h,
(6) Frame rate—40 Hz,
(7) The number of counts at the working section of the chirp—512,
(8) The number of chirps in one frame—*N* = 2048.

**Figure 5:**
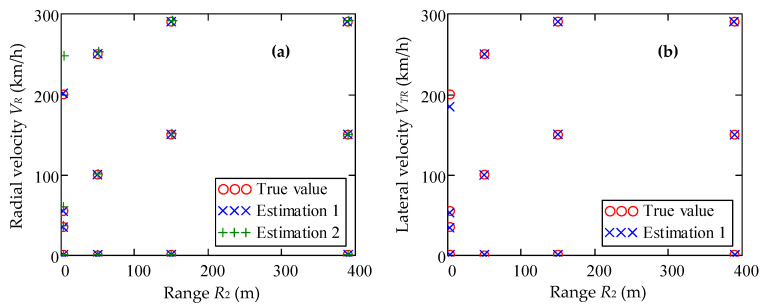
Estimation of motion parameters (**a**) *V_R_*, (**b**) *V_TR_* and *R*_2_: estimation 1 is the result of applying the developed algorithm, and estimation 2 is the application of the traditional 2D-FFT algorithm. Velocity vector angle *ψ* = 45°.

**Figure 6:**
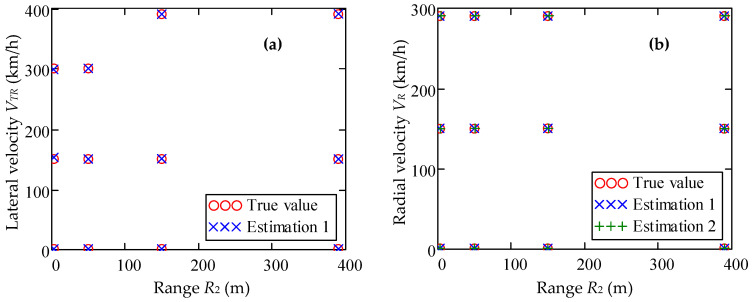
Estimation of motion parameters *V_R_*, *V_TR_* and *R*_2_: (**a**) velocity vector angle *ψ* = 90°, (**b**) *ψ* = 0°.

We tested radar targets with a given dynamic range of changes in the values of motion parameters—radial and transverse speeds, as well as range. The ratio between the radial and transverse components of the velocity was regulated by changing the angle ψ between the vector of the full velocity ***V*** (see Figure 2) and the radius vector ***R***_2_. In this case, the norm of the full-velocity vector ||***V***|| was fixed. In the computational experiment, the following motion modes were considered: *ψ* = 0°—strictly radial motion (moving away or approaching the target collinear to the radius vector ***R***_2_), *ψ* = 90°—strictly azimuthal (circular) movement, and *ψ* = 45°—motion in which the radial and transverse components of the velocity are equal. The results of the computational experiment for different angles, *ψ,* are shown in Figure 5 and Figure 6.

Consider the case *ψ* = 45°, when the radial and transverse velocity components are equal. Figure 5a, on the coordinate plane *V_R_*, *R*_2_ (radial speed-range) shows the results of the estimation (Estimation 2) of the radial speed and range to the target calculated using the traditional two-dimensional FFT method for the case *ψ* = 45°. Also given are estimates (Estimation 1) of the same parameter received via the developed algorithm. In the traditional method for estimating the parameters *V_R_* and *R*_2_, the terms Δ*f_B_*(*kT*_c_) and Δ*f_V_*(*kT*_c_) in relations Equation (25) and Equation (26) are assumed to be equal to zero, which leads to an increase in the error in estimating the motion parameters with decreasing range. 

It can be seen from Figure 5a that, in accordance with the results of theoretical analysis at short ranges (*R*_2_ = 3 m) and high target speeds (*V_R_* = 200 km/h), the estimate “Estimation 2” of the parameters *V_R_* and *R*_2_ has a large relative error in comparison with the estimate “Estimation 1”. The error of the traditional measurement method reaches 25% and 40% to estimate the speed and range, respectively. The error of the measurement method Estimation 1 of the coordinates of a point (*V_R_*, *R*_2_) does not exceed the value of *δR* × *δV* in the range-radial velocity measurement area [*R*_2_ × *V_R_*] = [3 m, 220 m] × [0 km/h, 220 km/h]. At the same time, at long range, both methods give the same estimate of the radial speed *V_R_*, which coincides with the true speed of the radar target. This is clearly seen on the right side of Figure 5a, where all three points of the *V_R_* value become indistinguishable.

In Figure 5b on the coordinate plane *V_TR_*, *R*_2_ (transverse speed-range) shows the results of Estimation 1 of the transverse component of the speed and range to the target calculated using the developed method for the case *ψ* = 45°. The error in determining the speed increases with decreasing range and increasing full speed, which is due to the approach of the coordinates of the point (*V_TR_*, *R*_2_) to the boundary of the region **Θ^(^**^3)^ Equation (35). A computational experiment shows that already at *R*_2_ ≥ 15 m, the error of the method for measuring the coordinates of a point (*V_TR_*, *R*_2_) does not exceed the value of *δR* × *δV* in the entire working range of the *V_TR_* and *R*_2_ parameters.

Figure 6 show the results of estimating the parameters of the target movement, calculated using the developed method for the case of strictly azimuthal (circular) movement *ψ* = 90° and strictly radial movement *ψ* = 0**°**, respectively. The analysis of the results of the computational experiment and the corresponding results of the theoretical analysis show that the relative error of the method for measuring the transverse velocity component *V_TR_* is greater than the error in measuring the radial component *V_R_*, but does not exceed 2% for the case *ψ* = 0° and 8% for the case *ψ* = 45° for *R*_2_ = 3 m (see Figure 5b and Figure 6a). 

**Figure 7:**
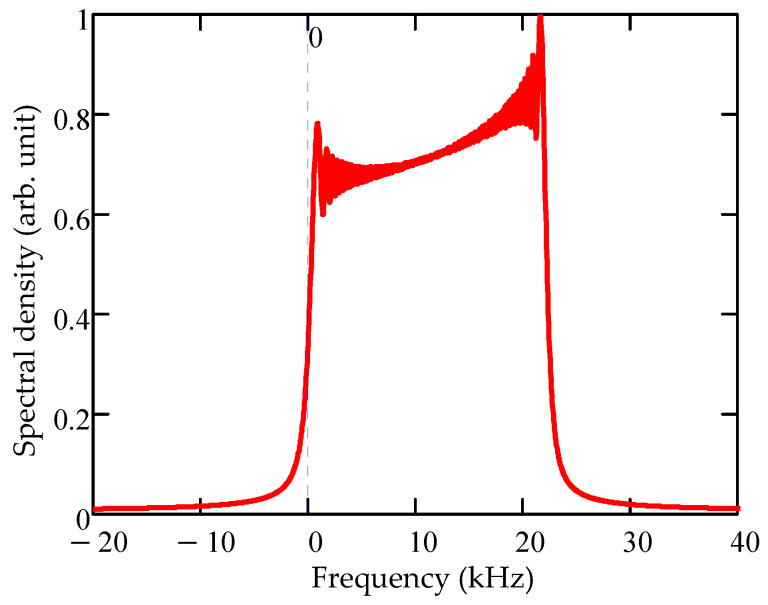
FFT conversion of radio echo signal in the slow time domain.

When assessing the motion parameters using the traditional method of two-dimensional FFT in the mode of strictly azimuthal (circular) target movement *ψ* = 90°, when the radial velocity component is absent, there is a large error in determining the value of *V_R_* at a short range and high object speed. Consider the physical interpretation of this result. This phenomenon is due to the temporal structure of the signal, which is a frequency-modulated signal. The Fourier spectrum of such a signal according to Equation (26) for *R*_2_ = 3 m and *V_TR_* = 290 km/h is shown in Figure 7. The maximum spectral component is at a frequency of 21.6 kHz, which corresponds to a radial speed of 153 km/h with a true value of *V_R_* = 0 km/h. At the same time, the estimate of the radial velocity, calculated using the developed algorithm, gives the value *V_R_* = 1.0 km/h and *V_TR_* = 287 km/h, which indicates the effectiveness of the algorithm.

An analysis of the results of estimating the range to a radar target with strictly radial movement *ψ* = 0° shows that the greatest error of the traditional FFT Estimation 2 method for measuring *R*_2_ is obtained at small ranges and high speeds of the observed object. So, with *R*_2_ = 3 m and *V_R_* = 290 km/h, Estimation 2 gives a value of 4.5 m, while Estimation 1 gives a value of 2.9 m, which is close to the true value.

Note that the computational experiment was carried out without the effect of noise on the radio signal. Therefore, the calculated results and the corresponding conclusions are correct for a large signal-to-noise ratio. In general, the results of numerical experiments agree well with the corresponding results of the theoretical analysis.

## 6. Conclusions

A rigorous mathematical, physically consistent model of the spatio-temporal structure of the echo signal of a radar operating in the active radar mode can be obtained using a quasi-relativistic vector transformation of Lorentz coordinates and time. This model is used to develop a software simulator of an echo-radio signal of a radar, which realizes the verification of the effectiveness of methods for assessing the parameters of vehicle movement by observation during one frame of a sample of an echo-radio signal of microwave LFMCW radars. The proposed model of the spatio-temporal structure of the echo signal of the LFMCW radar makes it possible to develop an algorithm for the simultaneous estimation of the velocity vector and the distance to the observed target, which remains operational in a wide range of changes in the parameters of the movement of vehicles. For the first time, an estimate of the radial and transverse components of the target velocity vector can be obtained by observing a number of data (sample) during one frame of the LFMCW radar sounding signal. For a 77 GHz microwave LFMCW radar with a sampling rate of about 55 MHz and a frame duration of 25 ms (frame rate 40 Hz), the range and speed measurement span with negligible noise level is from 3 to 400 m and from 1 to 290 km/h, respectively. The error of the proposed method for measuring the radial component of the speed and range of a radar target does not exceed the specified values of the resolution *δV* and *δR* in the entire specified working area of the range-radial velocity measurement. The error of the traditional method for measuring *V_R_* and *R*_2_ based on the two-dimensional Fourier transform is greater than the error of the proposed method. The relative error of the method for measuring the transverse velocity component *V_TR_* increases with decreasing range and increasing target speed, but does not exceed 2% for the case *ψ* = 0° and 8% for the case *ψ* = 45° with *R*_2_ = 3 m. The results of the computational experiment based on the developed rigorous mathematical model of the developed software simulator using the rigorous mathematical model of the LFMCW radar echo confirmed the corresponding theoretical analysis results. Thus, the previously set goal can be considered achieved.

For further work to validate the integrity of approaches to analyzing the performance of the developed algorithm by methods of computational or practical experiment, it is planned to develop an FMCW radar based on RF CMOS automotive transceiver TI AWR1243 and capture card DCA1000EVM (manufactured by Texas Instruments [47]) and make comparative test measurements.

Theoretically, for our further research, it is planned to tune the developed algorithm for estimating the parameters of movement in the presence of multiple radar targets and various kinds of radio interference. The mode of operation of the LFMCW radar in conditions of multiple radar targets and various kinds of radio interference is important in the practical use of the algorithm in modern systems of active driver assistance (ADAS—Advanced Driver Assistance System) to ensure the most comfortable and safe movement of the vehicle, including in the fully autonomous mode (unmanned) vehicle driving mode.
